# ﻿*Berberisjiuzhaigouensis* (Berberidaceae), a new riparian shrub from northern Sichuan, China

**DOI:** 10.3897/phytokeys.261.158475

**Published:** 2025-08-15

**Authors:** Hong-Li Pan, Yue Zhang, Jia-Hao Wang, De-Chang Meng, Chih-Chieh Yu

**Affiliations:** 1 Sichuan Academy of Forestry, Chengdu, 610081, Sichuan, China Sichuan Academy of Forestry Chengdu China; 2 Jiuzhaigou National Nature Reserve Administration Bureau, Aba Tibetan and Qiang Autonomous Prefecture, 623402, Sichuan, China Jiuzhaigou National Nature Reserve Administration Bureau Aba Tibetan and Qiang Autonomous Prefecture China; 3 College of Horticulture and Landscape Architecture, Zhongkai University of Agriculture and Engineering, Guangzhou, 510225, Guangdong, China Zhongkai University of Agriculture and Engineering Guangzhou China; 4 Grainger Bioinformatics Center, Field Museum of Natural History, Chicago, 60605, Illinois, USA Field Museum of Natural History Chicago United States of America

**Keywords:** *Berberis*, Jiuzhaigou, new species, phylogenomics, riparian flora

## Abstract

*Berberisjiuzhaigouensis* is herein described as a new deciduous species endemic to riparian habitats in Jiuzhaigou, northern Sichuan, China. Phylogenetic analyses based on complete plastome sequences and 322 nuclear loci consistently recover *B.jiuzhaigouensis* as a distinct and divergent lineage, genetically separated from all morphologically similar species and most closely related to *B.gilgiana*. Species delimitation analyses employing both topology-based (SODA) and substitution-based (bPTP, mPTP) frameworks further corroborate its taxonomic distinctiveness. This discovery highlights previously overlooked species diversity within *Berberis* in the Hengduan Mountains region.

## ﻿Introduction

The genus *Berberis* L. (Berberidaceae) comprises approximately 278 taxa in China, encompassing both evergreen and deciduous or semi-deciduous shrubs (Harber 2020), with new species continuing to be described in recent years (e.g., Li et al. 2022; Hajong et al. 2024). The majority of evergreen species are placed in Sect. Wallichianae, a lineage largely restricted to mainland Asia and adjacent subtropical islands. In contrast, deciduous species exhibit broader geographic distributions and occupy a wider range of ecological conditions (Harber 2020; Yu and Chung 2014). These two major lineages display contrasting biogeographic patterns across China and neighboring regions: deciduous taxa are typically associated with colder or more arid environments, such as the temperate zones of northern China, high-elevation regions of southwestern China, and the dry-hot valleys of the Hengduan Mountains (HDM), whereas evergreen taxa are generally confined to mid-elevation zones or to the understory of humid subtropical montane systems, including Wuyi Shan and Nanling (Ying 2011).

Deciduous *Berberis* species exhibit markedly higher taxonomic richness than their evergreen counterparts in China, particularly in the HDM region (Li et al. 2022). However, the northeastern margin of the HDM, where it meets the Qinling Range, exhibits relatively low *Berberis* species diversity (Harber 2020). Only about 20 species have been recorded from southern Gansu and the Hongyuan – Jiuzhaigou area in northern Sichuan – comprising approximately 7% of China’s *Berberis* species diversity (Harber 2020). Most of these are deciduous, with only three known evergreen species – *B.soulieana* C. K. Schneid., *B.bergmanniae* C. K. Schneid., and *B.verruculosa* Hemsl. & E. H. Wilson – recorded in the region. The floristic inventory in the Jiuzhaigou area remains incomplete due to the region’s complex topography.

During a field expedition in 2023 to the northeastern mountains of Jiuzhaigou County, we discovered an undescribed *Berberis* taxon. No corresponding records were found in regional herbaria. The population occupies a riparian habitat at approximately 1600 meters elevation. The species exhibits highly distinctive foliar morphology unlike any known species in the northern temperate or adjacent floras. Some individuals bear narrow, linear-entire leaves, while others develop broader, willow-like blades. The mature stems are purplish-red (see detailed color comparisons in Li et al. 2022). Based on leaf morphology, the unknown taxon most closely resembles *B.gilgiana* Fedde and *B.caroli* C. K. Schneid., but differs markedly in inflorescence and floral traits (Suppl. material 1). The taxon is locally abundant but is currently known only from a single site. Its linear leaves and slender, flexible shoots appear to be adaptations to the riparian habitat, which likely experiences periodic flooding during the monsoon season.

To assess its taxonomic distinctiveness, we conducted molecular phylogenetic analyses using whole plastome sequences and nuclear loci. Rooted by an evergreen species, the results consistently placed the new taxon as a genetically distinct lineage, divergent from all sampled morphological relatives. Together with its morphological (Table 1) and ecological distinctiveness, these data support recognition of this taxon as a new species, which we describe as *Berberisjiuzhaigouensis*.

**Table 1.** Morphological comparison of *Berberisjiuzhaigouensis* and the selected species.

	*B.jiuzhaigouensis*	*B.gilgiana*	*B.salicaria*	*B.chinensis*	*B.caroli*	*B.purdomii*
Inflorescence	a fascicle, sub-umbel, solitary or 6–8-flowered	a spike-like raceme 10 to 25-flowered	a spike-like raceme, ca. 25-flowered	a raceme, sometimes sub-umbellate at tip and or partly paniculate, 8 to 30-flowered	a spike-like raceme	a spike-like raceme, 15 to 25-flowered
Leaf shape	lanceolate, obovate-lanceolate or narrowly elliptic	lanceolate., obovate-lanceolate or narrowly elliptic	lanceolate	oblanceolate to narrowly oblanceolate	narrowly oblanceolate, spatulate-oblanceolate, obovate-lanceolate or narrowly elliptic	obovate, obovate-spatulate
Leaf blade abaxially	glabrous	pubescent	glabrous	Glabrous	glabrous	glabrous
Leaf margin	entre	entire,, rarely spinulose with 1–4 teeth on each side	spinose with 15–40 teeth on each side inconspicuous teeth on each side	entire	entire	spinose with 3–9 teeth on each side
Shape of leaf apex	acute	acute	subacuminate	acuminate or acute, mucronate	obtuse, rarely acute, mucronate	acute
Coloration of mature stem	brown or purplish-red	reddish or purplish-brown	yellowish-brown	pale reddish brown	purplish	purplish red
No. of spines below short branch	solitary or absent	solitary or 3-fid	solitary, rarely 2 or 3-fid	absent or solitary, sometimes 3-fid	solitary	solitary
No. ovules	2, 3	2	2	1	2	2

## ﻿Materials and methods

### ﻿Sampling, molecular data, and phylogenetic reconstruction

Seven deciduous species exhibiting potential phylogenetic and morphological similarity to *Berberisjiuzhaigouensis* were selected for phylogenetic reconstruction (Suppl. material 1), along with *B.wallichiana* C. K. Schneid., an evergreen taxon designated as the outgroup. Among the selected species, *B.gilgiana*, *B.chinensis*, *B.caroli*, *B.salicaria*, and *B.purdomii* were included due to their similar leaf morphology and geographic proximity to the Jiuzhaigou region. These species are generally considered part of the northern temperate clade of *Berberis*. In contrast, *B.wilsoniae* and *B.ninglangensis* were selected to represent a sister clade to this group, providing broader phylogenetic context for comparison. Genomic DNA of each species was extracted either from silica gel–dried leaf tissue or from herbarium specimens. Purified genomic DNA was sheared and used to construct short-insert libraries (500 bp) following the manufacturer’s protocol. Libraries were quantified with an Agilent 2100 Bioanalyzer and sequenced on the Illumina NovaSeq 6000 platform at Novogene Co. Demultiplexed raw reads were trimmed using *illumiprocessor* v2.0.9, and the resulting clean reads were assembled into complete plastomes using *GetOrganelle* v2.7.2 (Jin et al. 2020), employing a seed-and-extend algorithm with the plastome of *B.amurensis* (GenBank accession ID: MH926107.1) as reference. The assembled plastome sequences were annotated and manually checked using *Geneious* v9.0.5 (Kearse et al. 2012). We performed a Maximum Likelihood (ML) analysis using the “ML + Thorough bootstrap” strategy implemented in the “RAxML-HPC2 Workflow” on CIPRES (https://www.phylo.org/). To optimize phylogenetic resolution and branch support, we employed complete chloroplast genomes, excluding one of the two inverted repeat (IR) regions to reduce computational load. Accordingly, the ML analysis was performed with the following settings: 10 alternative runs with distinct starting trees, 1,000 bootstrap iterations, GTRGAMMA model, and default settings for the remaining parameters. *B.wallichiana* was selected as the outgroup.

For nuclear phylogenetic inference, we assembled 332 gene loci from trimmed reads using *HybPiper* v1.3.1 (Johnson et al. 2019), with the Angiosperms353 bait set, using *Berberissibirica* as the reference target (Baker et al. 2022). Each locus was recovered as a supercontig, comprising both exons and flanking intronic regions. For concatenated phylogenetic analysis, aligned sequences from all loci were concatenated using a custom bash script and analyzed with *IQ-TREE* multicore v2.3.3 (Nguyen et al. 2015). Model selection was performed using *ModelFinder* with the ‘-m MFP’ option (Kalyaanamoorthy et al. 2017), followed by maximum likelihood inference. Nodal support was assessed with 1,000 ultrafast bootstrap replicates (Hoang et al. 2018) and Shimodaira–Hasegawa-like approximate likelihood ratio tests (SH-aLRT; Anisimova et al. 2011). For species tree estimation under the multispecies coalescent (MSC) model, individual gene trees for all loci were each inferred using the same *IQ-TREE* pipeline, and the species tree was summarized using *ASTRAL* v5.7.7 (Mirarab and Warnow 2015) under default parameters. The resulting trees – plastome ML topology with bootstrap support, concatenated nuclear ML topology with ultrafast bootstrap values, and *ASTRAL* species tree with local posterior probabilities (localPP) – were visualized using *FigTree* v1.4.2.1 (Rambaut 2010).

### ﻿Inference of species boundaries using topology- and substitution-based models

We used the *ASTRAL* species tree to evaluate the species hypothesis of *Berberisjiuzhaigouensis* and related taxa. Specifically, we implemented species boundary delimitation using *ASTRAL* (*SODA* v1.0.2; Rabiee and Mirarab 2021), a coalescent-based method that performs species delimitation using gene tree topologies alone. *SODA* incorporates a polytomy test within the *ASTRAL* framework to statistically evaluate whether each internal branch of the species tree has significant quartet support. Branches lacking support are collapsed, producing a delimitation hypothesis without requiring branch length or sequence divergence data. Analyses were conducted using default parameters and a significance threshold of 0.001. We applied two variants of the Poisson Tree Processes (PTP) model, which delimit species based on the number of substitutions inferred along the branches of a phylogenetic input tree. The Bayesian implementation (*bPTP*; Zhang et al. 2013) was conducted via the online server (https://species.h-its.org/ptp/), while the multi-rate maximum-likelihood version (*mPTP*; Kapli et al. 2017) was executed at https://mptp.h-its.org/, using a p-value cutoff of 0.001. Unlike *SODA*, both PTP methods assume that branching events follow distinct Poisson processes for speciation versus within-species coalescence, using either a single or multiple substitution rate model. *mPTP* applies dynamic programming to optimize species partitioning under a likelihood framework, whereas *bPTP* employs Bayesian inference to estimate posterior support for putative species boundaries. To minimize potential biases associated with distant outgroups, *B.wallichiana* was excluded from all analyses, following established recommendations. Given the distinct assumptions and model frameworks of *SODA*, *bPTP*, and *mPTP*, we compared results across methods to preliminarily assess the robustness and congruence of species boundaries inferred from multi-locus phylogenomic data.

## ﻿Results and discussion

### ﻿Phylogeny and inference of species boundaries

**Figure 1.**
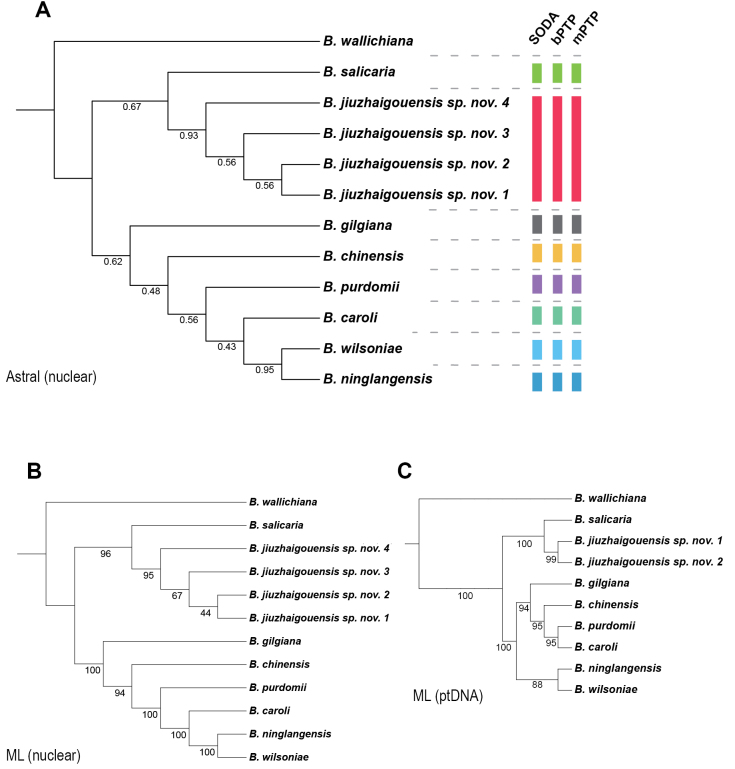
Species delimitation results for *Berberisjiuzhaigouensis* and related deciduous taxa based on nuclear and plastid phylogenies. **A.** Rooted *ASTRAL* species tree inferred from 332 nuclear gene trees, with local posterior probabilities shown on branches; **B.** Maximum likelihood tree based on the concatenated 332 nuclear gene sequences, with bootstrap support values indicated; **C.** Maximum likelihood tree based on complete plastome (ptDNA) sequences, with bootstrap support values indicated. Species boundaries were inferred using three methods: *SODA* (topology-based), *bPTP*, and *mPTP* (substitution-based). In panel **A**, alternative species delimitation hypotheses are represented by distinct colors.

Both plastid and nuclear phylogenies consistently recover *Berberisjiuzhaigouensis* as a distinct lineage (Fig. 1). Across all nuclear datasets, the four sampled individuals form a well-supported, reciprocally monophyletic clade in both concatenated ML and *ASTRAL* species trees, with *B.salicaria* consistently inferred as the sister taxon. This relationship is supported by high plastome bootstrap values and moderate local posterior probabilities in the *ASTRAL* analysis.

Species delimitation results were congruent across methods. The coalescent-based *SODA* framework identified *B.jiuzhaigouensis* as an independently evolving lineage, with strong support from quartet scores in the *ASTRAL* tree. Substitution-based methods (*bPTP*, *mPTP*) yielded consistent delimitations, reinforcing the inference of lineage distinctiveness under alternative model assumptions. Together, these results provide convergent evidence for the species status of *B.jiuzhaigouensis*, supported by both tree topology and species delimitation frameworks grounded in divergent theoretical foundations.

### ﻿Taxonomic treatment

#### ﻿ Berberis jiuzhaigouensis

Taxon classificationPlantaeRanunculalesBerberidaceae

H.L.Pan, D.C.Meng & C.C.Yu sp. nov.

09F43C00-6B0B-5721-99DB-9F98C719EA30

urn:lsid:ipni.org:names:77367213-1

Fig. 2

##### Diagnosis.

Berberisjiuzhaigouensis is morphologically similar to *B.gilgiana* and *B.salicaria*, particularly in leaf shape. However, it can be readily distinguished by its glabrous abaxial leaf surface – contrasting with the distinctly pubescent midrib of *B.gilgiana* (Suppl. material 1: fig. S1A) – and by its fascicled to sub-umbellate inflorescences, in contrast to the spike-like racemes characteristic of both *B.gilgiana* and *B.salicaria*.

##### Type.

China • Sichuan, Aba Tibetan and Qiang Autonomous Prefecture, Jiuzhaigou County, Wujiao Town, Shengnanxin Village, around the point 33.08206737°N,104.17426166°E, alt. 1636 m, 16 Apr. 2023, YCC415 (holotype: HITBC0127178!; Suppl. material 2)

##### Description.

Shrubs, deciduous, to 1.5 m tall; mature stems brown or purplish-red, terete; spines 3-fid, often solitary towards apex of stems, 0.2–1 cm, weak. Petiole almost absent; leaf blade abaxially pale green, adaxially green, lanceolate, obovate-lanceolate, or narrowly elliptic, 2.5–6 × 0.2–1.5 cm; lateral veins and reticulation partially raised abaxially, inconspicuous adaxially; base attenuate; margin entire; apex acute. Inflorescence a fascicle, sub-umbel, solitary, or 6–8-flowered, ca. 3 cm including peduncle 1 cm; pedicel 2 cm, bracteoles triangular. Sepals in 3 whorls, outer sepals narrowly elliptic 3–3.5 × 1.5–2 mm, median sepals elliptic-obovate 4.5–5 × 3–3.5 mm; inner sepals elliptic-orbicular 5–5.5 × 3.5–4 mm; petals obovate or orbicular-obovate, 5–6 × 6–7 mm, base distinctly clawed; glands separate, elliptic or obovate, 0.75–1 mm, apex entire or incised. Stamens 2.5–3 mm; anther connective truncate. Ovules 2, 3. Berry red, ellipsoid 8–9 mm × ca. 5 mm, estylose.

**Figure 2.**
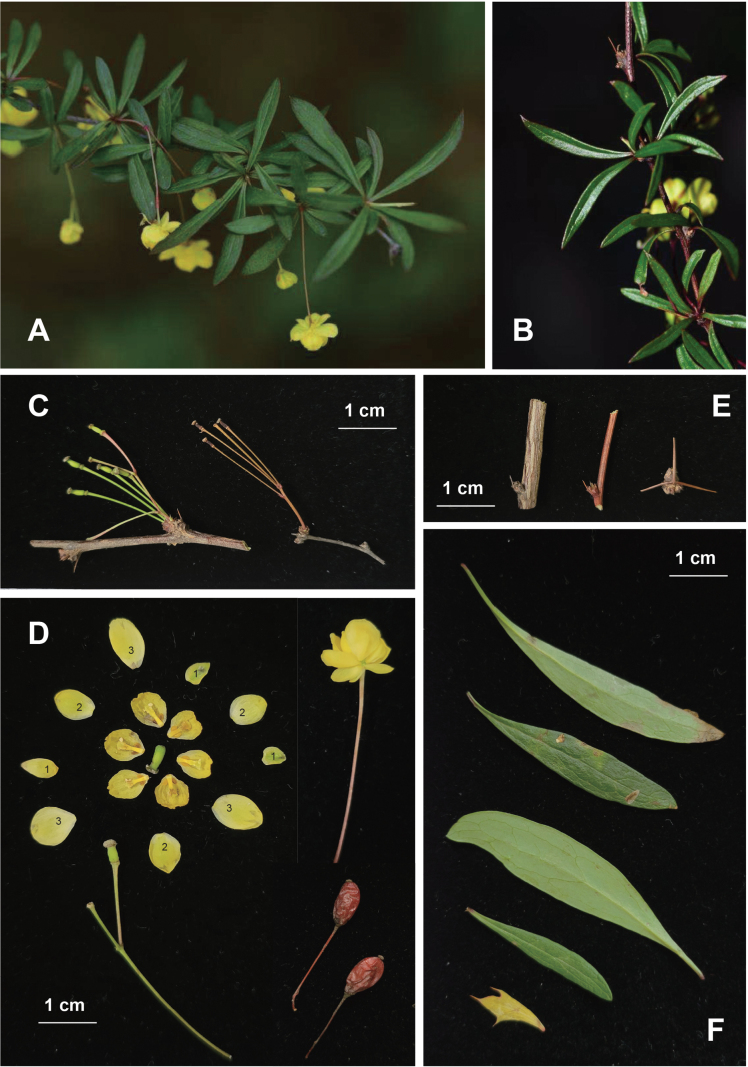
*Berberisjiuzhaigouensis* sp. nov. **A.** Flowering branches; **B.** Streamlined, narrowly elliptic leaf blades with purplish-brown branches; **C.** Inflorescence types (clustered on the left, sub-umbellate on the right); **D.** Reproductive structures, with sepal numbering indicating their positional order; **E.** Spines on short shoots; **F.** Leaf morphology.

##### Phenology.

*Berberisjiuzhaigouensis* has been collected in flower in April and in fruit between May and October.

##### Distribution and habitat.

*Berberisjiuzhaigouensis* is currently known only from the type locality. It has been collected from ravine habitats and valley slopes at elevations ranging from approximately 1,600 to 2,000 m a.s.l.

## Supplementary Material

XML Treatment for Berberis jiuzhaigouensis

## ﻿Additional information

### Use of AI

No use of AI was reported.

### Author ORCIDs

Hong-Li Pan https://orcid.org/0000-0003-0943-2398

Yue Zhang https://orcid.org/0009-0004-1222-8747

De-Chang Meng https://orcid.org/0000-0002-1049-1232

Chih-Chieh Yu https://orcid.org/0000-0002-4647-6309

### Data availability

All of the data that support the findings of this study are available in the main text or Supplementary Information.

## Supplementary materials

Supplementary material 1 Color plate of *B.gilgiana*, *B.chinensis*, *B.caroli*, *B.purdomii* Data type pdf Explanation note **fig. S1. A.***B.gilgiana*, accompanied by a magnified view highlighting the pubescent leaf surface; **B.***B.chinensis*; **C.***B.caroli*; **D.** a young shoot of *B.purdomii*. These species most closely resemble B.jiuzhaigouensis in leaf shape and general morphology, but all differ from it by possessing racemose or spike-like racemose inflorescences. https://binary.pensoft.net/file/1395502This dataset is made available under the Open Database License (http://opendatacommons.org/licenses/odbl/1.0/). The Open Database License (ODbL) is a license agreement intended to allow users to freely share, modify, and use this Dataset while maintaining this same freedom for others, provided that the original source and author(s) are credited.Hong-Li Pan, Yue Zhang, Jia-Hao Wang, De-Chang Meng, Chih-Chieh Yu

Supplementary material 2 Image of the holotype of *Berberisjiuzhaigouensis* Data type pdf https://binary.pensoft.net/file/1395503This dataset is made available under the Open Database License (http://opendatacommons.org/licenses/odbl/1.0/). The Open Database License (ODbL) is a license agreement intended to allow users to freely share, modify, and use this Dataset while maintaining this same freedom for others, provided that the original source and author(s) are credited.Hong-Li Pan, Yue Zhang, Jia-Hao Wang, De-Chang Meng, Chih-Chieh Yu

Supplementary material 3 Supplementary table Data type xlsx https://binary.pensoft.net/file/1395504This dataset is made available under the Open Database License (http://opendatacommons.org/licenses/odbl/1.0/). The Open Database License (ODbL) is a license agreement intended to allow users to freely share, modify, and use this Dataset while maintaining this same freedom for others, provided that the original source and author(s) are credited.Hong-Li Pan, Yue Zhang, Jia-Hao Wang, De-Chang Meng, Chih-Chieh Yu
