# Supplementary figures and images for: ﻿Berberisjiuzhaigouensis (Berberidaceae), a new riparian shrub from northern Sichuan, China
Source: PhytoKeys. 2025 Aug 15;261:165–74. doi: 10.3897/phytokeys.261.158475 (PMC12374172; doi:10.3897/phytokeys.261.158475)

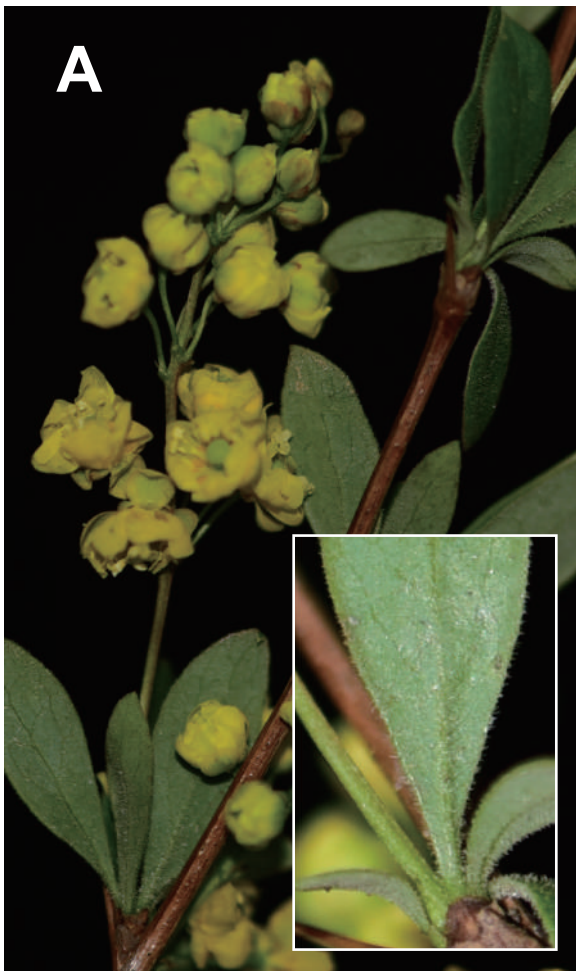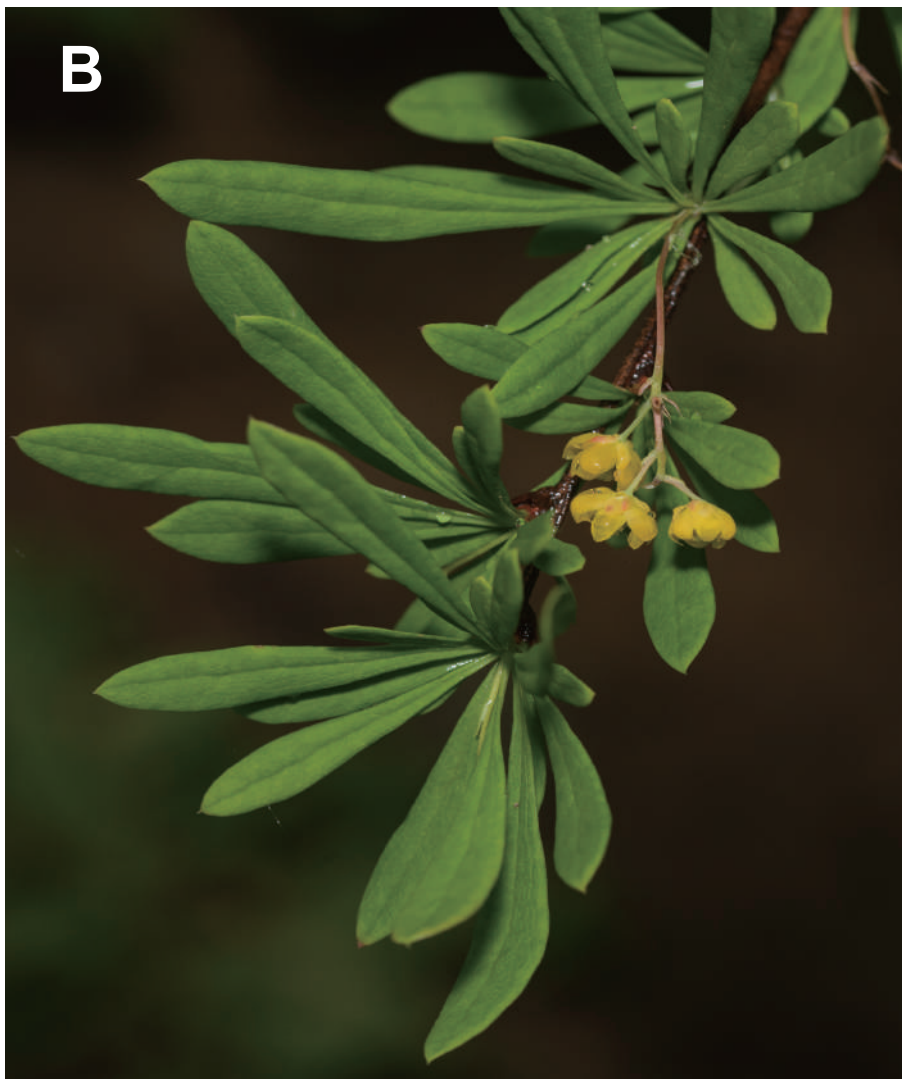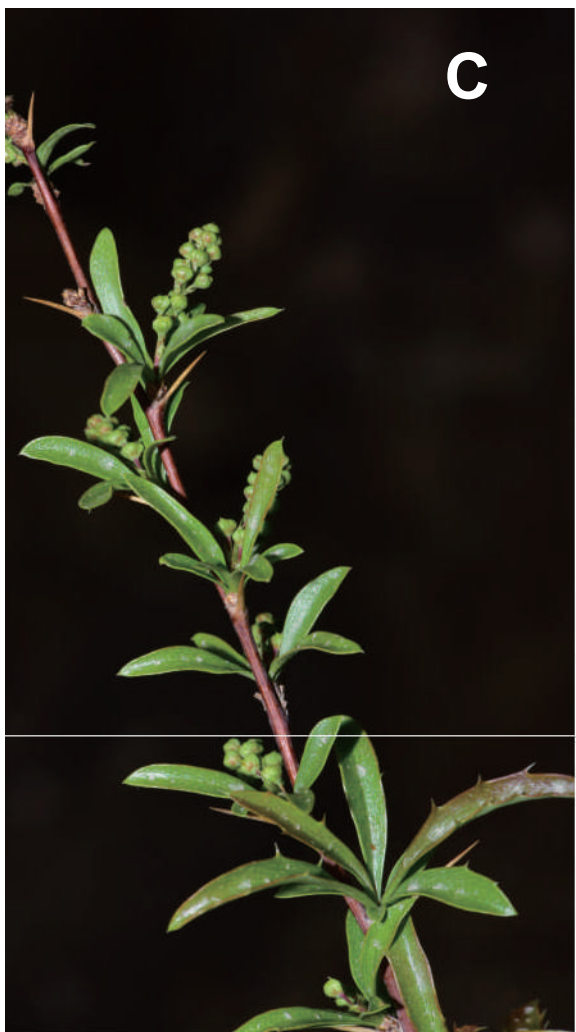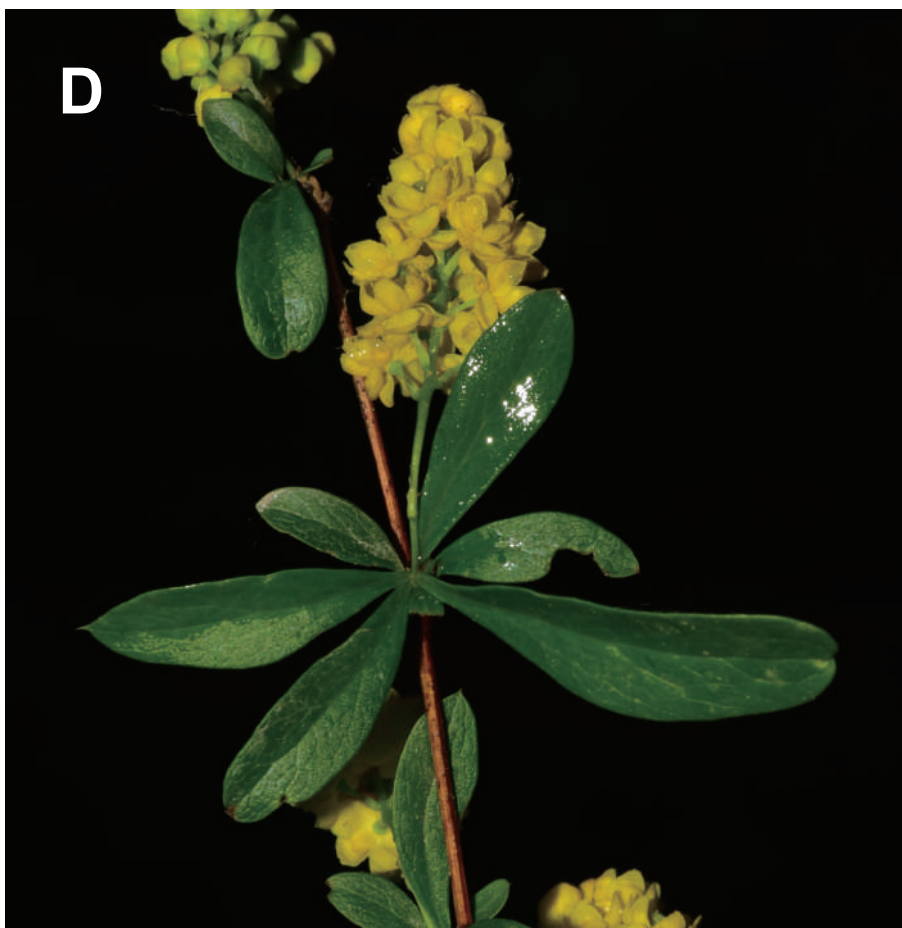

Supplement: Supplementary material 1 — Color plate of B.gilgiana, B.chinensis, B.caroli, B.purdomii [file phytokeys-261-165_article-158475__-s001.pdf]
