# Supplementary material for: ﻿Berberisjiuzhaigouensis (Berberidaceae), a new riparian shrub from northern Sichuan, China
Source: PhytoKeys. 2025 Aug 15;261:165–74. doi: 10.3897/phytokeys.261.158475 (PMC12374172; doi:10.3897/phytokeys.261.158475)
Supplement: Supplementary material 2 — Image of the holotype of Berberisjiuzhaigouensis [file phytokeys-261-165_article-158475__-s002.pdf]

Herbarium of Xishuangbanna
Tropical Botanical Garden
Chinese Academy of Sciences
No. 211918

中国科学院西双版纳热带植物园标本馆（HITBC）
HITBC0127178

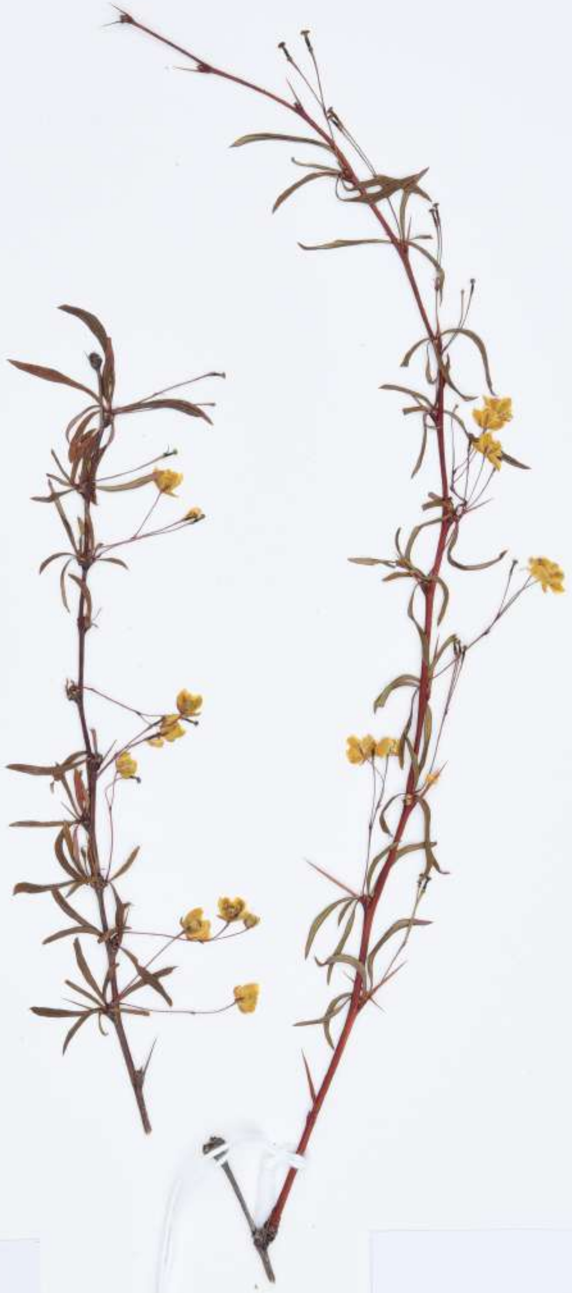

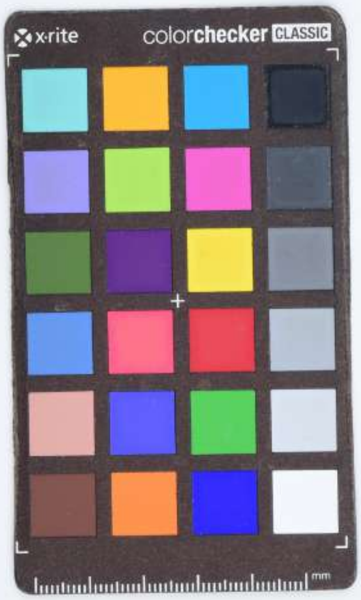

B. jiuzhaigouensis
定名人(日期)： holotype

材料袋
HITBC
中国科学院西双版纳热带植物园标本馆
Herbarium of Xishuangbanna Tropical Botanical Garden,CAS

YCC415

Flora of China

采集号 Coll. No.: YCC415　采集日期 Coll. Date: 16. Apr. 2023
采集人 Collector: Hong-Li Pan, Yue Zhang, Jia-Hao Wang, De-Chang Meng
采集地 Locality: Sichuan, Aba Tibetan and Qiang Autonomous Prefecture, [illegible], Wujiao Town, [illegible] Village
经纬度 Long./Lat.: 33.08206737°N，104.17426166°E　海拔 Alt.: 1636 m
生境 Habitat: riparian region, stream side
习性 Habit: evergreen　体高 Height: m　胸径 DBH: cm
性状 Character:
中文名 Chinese Name: 九寨沟小檗　科名 Family: Berberidaceae
学名 Sci. Name: *Berberis jiuzhaigouensis*
备注 Note: Holotype
